# Supplementary material for: Understanding cervical cancer awareness in hard-to-reach areas of Bangladesh: A cross-sectional study involving women and household decisionmakers
Source: PLoS One. 2024 Aug 9;19(8):e0304396. doi: 10.1371/journal.pone.0304396 (PMC11315347; doi:10.1371/journal.pone.0304396)
Supplement: S1 Table — (DOCX) [file pone.0304396.s001.docx]

**S1 Table. Knowledge score of cervical cancer risk factors and symptoms**

|  | Women (n=600) | Decisionmakers (n=1200) | |
| --- | --- | --- | --- |
|  | Female respondents (n=486), n (%) | Husband (n=406), n (%) | Mother-in-law respondents (n=445), n (%) |
| Knowledge scores |  |  |  |
| Risk factors for cervical cancer | Median:6 IQR: (4 -8) | Median:6 IQR: (4 - 8) | Median:6 IQR: (3 - 8) |
| Low knowledge | 205 (42.1) | 172 (42.4) | 218 (48.9) |
| High knowledge | 281 (57.8) | 234 (57.6) | 227(51.0) |
|  | Female respondents (n=600), n (%) | Husband (n=600), n (%) | Mother-in-law respondents (n=600), n (%) |
| Symptoms of cervical cancer | Median:8 IQR: (4 - 10) | Median:7 IQR: (4 - 9) | Median:8 IQR: (4 - 10) |
| Low knowledge | 265 (44.2) | 262 (43.7) | 299 (49.8) |
| High knowledge | 335 (55.8) | 338 (56.3) | 301 (50.2) |
