# Supplementary material for: Understanding cervical cancer awareness in hard-to-reach areas of Bangladesh: A cross-sectional study involving women and household decisionmakers
Source: PLoS One. 2024 Aug 9;19(8):e0304396. doi: 10.1371/journal.pone.0304396 (PMC11315347; doi:10.1371/journal.pone.0304396)
Supplement: S1 Questionnaire — (DOCX) [file pone.0304396.s002.docx]

**S1 Questionnaire. AWACAN Questionnaire**

| COUNTRY    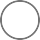 1 = Bangladesh  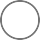 2 = India  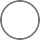 3 = Slovakia  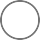 4 = Uganda |
| --- |
| PARTICIPANT'S GROUP  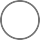 1 = Intervention  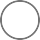 2 = Control |
| RESPONDENT CATEGORY  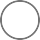 1 = Women  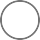 2 = Husband  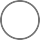 3 = Mother-in-Law/ Decision Maker |
| RESPONDENT ID |
| DEMOGRAPHIC QUESTIONS |
| A. AGE |

| B. CURRENT RELATIONSHIP STATUS?  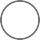 3 = Currently married  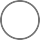 5 = Divorced  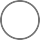 6 = Widowed  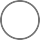 8 = Refused to answer/ Not provided |
| --- |
| C. SEX  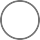 1 = Male  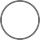 2 = Female |
| D. HIGHEST LEVEL OF EDUCATION?  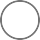 0 = No education  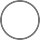 1 = Not completed primary  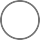 2 = Completed primary  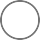 3 = Completed secondary  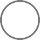 4 = Completed higher secondary  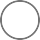 5 = Completed bachelors  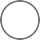 6 = Completed masters |
| E. VILLAGE/ CHAR OF LIVING |
| F. RELIGION  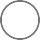 1 = Islam  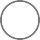 2 = Hindu  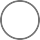 3 = Buddhist  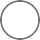 4 = Christian  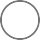 99 = Others |

| G. MONTHLY EXPENDITURE IN BDT S1 Questionnaire. AWACAN Questionnaire  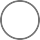 1 = 0 - 5000  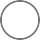 2 = 5001 - 10000  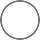 3 = 10001 - 20000  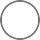 4 = 20001 - 30000  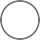 5 = > 30000 |
| --- |
| H. OCCUPATION |
| I. MOBILE NUMBER |
| J. DISTRICT NAME  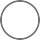 1 = Gaibandha  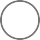 2 = Kurigram  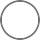 3 = Satkhira  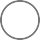 4 = Bogura  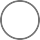 5 = Sirajganj |
| BASELINE UPTAKE QUESTIONS |
| C1. HAS THE HEALTHCARE WORKER EVER TESTED YOU FOR CERVICAL CANCER?  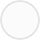 1 = Yes  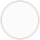 2 = No  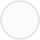 88 = (Do not read) Don't know  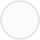 99 = (Do not read) Refused |

| C2. WHEN WAS YOUR LAST TEST FOR CERVICAL CANCER?  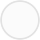 1 = <1 year ago  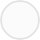 2 = 1 - 2 years ago  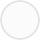 3 = 3 - 5 years ago  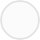 4 = >5 years ago  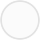 88 = (Do not read) Don't know  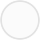 99 = (Do not read) Refused |
| --- |
| C3. WHAT WAS THE RESULT OF YOUR LAST TEST FOR CERVICAL CANCER?  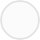 1 = Did not receive result  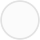 2 = Normal/ negative  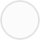 3 = Abnormal/ positive  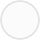 4 = Suspect cancer  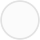 5 = Inconclusive  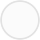 88 = (Do not read) Don't know  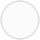 99 = (Do not read) Refused |
| C4. DID YOU HAVE ANY FOLLOW-UP VISITS BECAUSE OF YOUR LAST TEST RESULT?  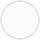 1 = Yes  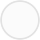 2 = No  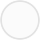 88 = (Do not read) Don't know  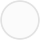 99 = (Do not read) Refused |
| C5. DID YOU HAVE ANY TREATMENT TO YOUR CERVIX BECAUSE OF YOUR LAST TEST RESULT?  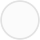 1 = Yes  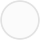 2 = No  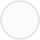 88 = (Do not read) Don't know  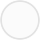 99 = (Do not read) Refused |
| AFRICAN WOMEN AWARENESS OF CANCER (AWACAN) - CERVICAL CANCER TOOL ADAPTED VERSION |
| DECISION-MAKING |

| 0. WHO IN YOUR HOUSEHOLD USUALLY HAS THE FINAL SAY IN YOUR OWN HEALTH?  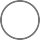 1 = Myself  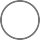 2 = Partner  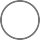 3 = Myself and partner jointly  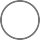 4 = Someone else in household  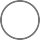 5 = Myself and someone else jointly  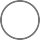 99 = Decision not taken/ question does not apply |
| --- |
| SECTION 1. CERVICAL CANCER AWARENESS MEASURE |
| Q 1. HAVE YOU EVER HEARD OF CERVICAL CANCER? [IF "NO", SKIP TO SECTION 3](https://ee.kobotoolbox.org/preview/i/%E0%A6%86%E0%A6%AA%E0%A6%A8%E0%A6%BF%20%E0%A6%95%E0%A6%BF%20%E0%A6%95%E0%A6%96%E0%A6%A8%E0%A6%93%20%E0%A6%9C%E0%A6%B0%E0%A6%BE%E0%A7%9F%E0%A7%82%20%E0%A6%95%E0%A7%8D%E0%A6%AF%E0%A6%BE%E0%A6%A8%E0%A7%8D%E0%A6%B8%E0%A6%BE%E0%A6%B0%E0%A7%87%E0%A6%B0%20%E0%A6%AC%E0%A6%BF%E0%A6%B7%E0%A7%9F%E0%A7%87%20%E0%A6%B6%E0%A7%81%E0%A6%A8%E0%A7%87%E0%A6%9B%E0%A7%87%E0%A6%A8)  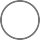 1 = Yes (  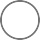 0 = No |
| Q 2. DO YOU KNOW OF ANY FAMILY MEMBERS, FRIENDS OR NEIGHBORS WHO HAVE/HAD CERVICAL CANCER?  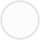 1 = Yes  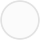 0 = No |
| Q 3. COULD YOU PLEASE NAME AS MANY THINGS AS YOU CAN THINK OF THAT COULD INCREASE ANY WOMAN'S  CHANCES OF GETTING CERVICAL CANCER?" |
| SECTION 2. KNOWLEDGE OF RISK FACTOR |
| Q 2A. COULD ANY OF THE FOLLOWING INCREASE ANY WOMAN'S CHANCES OF GETTING CERVICAL CANCER? |
| Q 4. GETTING A SEXUALLY TRANSMITTED INFECTION CALLED THE HUMAN PAPILLOMAVIRUS (HPV)  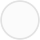 1 = Yes  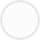 0 = No  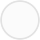 99 = Don't know |

| Q 5. HIV/AIDS  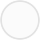 1 = Yes  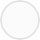 0 = No  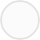 99 = Don't know |
| --- |
| Q 6. BEING INFECTED WITH OTHER SEXUALLY TRANSMITTED DISEASES (OTHER THAN HIV OR HUMAN PAPILLOMAVIRUS)  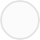 1 = Yes  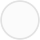 0 = No  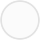 99 = Don't know |
| Q 7. USING BIRTH CONTROL PILLS/FAMILY PLANNING FOR MORE THAN 5 YEARS  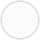 1 = Yes  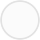 0 = No  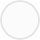 99 = Don't know |
| Q 8. USING CONDOMS  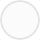 1 = Yes  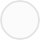 0 = No  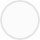 99 = Don't know |
| Q 9. HAVING UNPROTECTED SEX  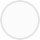 1 = Yes  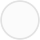 0 = No  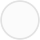 99 = Don't know |
| Q 10. SMOKING ANY CIGARETTES AT ALL  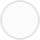 1 = Yes  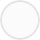 0 = No  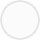 99 = Don't know |
| Q 11. INSERTING HERBS/CREAMS/OBJECTS INTO THE VAGINA  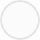 1 = Yes  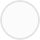 0 = No  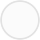 99 = Don't know |

| Q 12. HAVING A SEXUAL PARTNER WHO IS NOT CIRCUMCISED  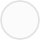 1 = Yes  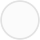 0 = No  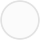 99 = Don't know |
| --- |
| Q 13. HAVING SEX AT A YOUNG AGE (BEFORE 17 YEARS)  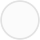 1 = Yes  0 = No  99 = Don't know |
| Q 14. POOR PERSONAL HYGIENE E.G. NOT WASHING ONE'S VAGINA WELL, STAYING FOR LONG WITHOUT BATHING OR  WEARING WET  1 = Yes  0 = No  99 = Don't know |
| Q 15. GIVING BIRTH TO THREE OR MORE CHILDREN  1 = Yes  0 = No  99 = Don't know |
| Q 16. HAVING MANY SEXUAL PARTNERS  1 = Yes  0 = No  99 = Don't know |
| Q 17. NOT GOING FOR REGULAR SCREENING/TESTING FOR CERVICAL CANCER  1 = Yes  0 = No  99 = Don't know |
| Q 18. BEWITCHED/WITCHCRAFT/EVIL SPIRITS  1 = Yes  0 = No  99 = Don't know |

| SECTION 3. KNOWLEDGE OF SYMPTOMS |
| --- |
| Q 19. COULD YOU PLEASE NAME AS MANY SYMPTOMS OR SIGNS OF CERVICAL CANCER AS YOU CAN THINK OF? |
| Q. CAN YOU TELL ME IF YOU THINK THE FOLLOWING COULD BE SIGNS OF SOMETHING SERIOUS OR THAT SOMETHING IS WRONG SUCH AS CERVICAL CANCER? |
| Q 20. VAGINAL BLEEDING BETWEEN MENSTRUAL PERIODS  1 = Yes  0 = No  99 = Don't know |
| Q 21. PERSISTENT LOWER BACK PAIN  1 = Yes  0 = No  99 = Don't know |
| Q 22. A PERSISTENT SMELLY VAGINAL DISCHARGE  1 = Yes  0 = No  99 = Don't know |
| Q 23. DISCOMFORT OR PAIN DURING SEX  1 = Yes  0 = No  99 = Don't know |

| Q 24. MENSTRUAL PERIODS THAT ARE LONGER OR HEAVIER THAN USUAL  1 = Yes  0 = No  99 = Don't know |
| --- |
| Q 25. PERSISTENT DIARRHEA  1 = Yes  0 = No  99 = Don't know |
| Q 26. VAGINAL BLEEDING AFTER MENOPAUSE [EXPLANATION]: MENOPAUSE IS WHEN A WOMAN'S PERIODS HAVE  STOPPED PERMANENTLY  1 = Yes  0 = No  99 = Don't know |
| Q 27. PERSISTENT LOWER ABDOMINAL/PELVIC PAIN  1 = Yes  0 = No  99 = Don't know |
| Q 28. VAGINAL BLEEDING DURING OR AFTER SEX  1 = Yes  0 = No  99 = Don't know |

| Q 29. BLOOD IN URINE OR STOOL (FAECES) [EXPLANATION]: BLOOD IN PEE/WEE OR  1 = Yes  0 = No  99 = Don't know |
| --- |
| Q 30. UNEXPLAINED WEIGHT LOSS  1 = Yes  0 = No  99 = Don't know |
| Q 31. ITCHING IN THE VAGINA  1 = Yes  0 = No  99 = Don't know |
| SECTION 4: HELP-SEEKING BEHAVIOUR |
| Q 32. IF YOU HAD A SYMPTOM COMING FROM YOUR CERVIX OR MOUTH OF YOUR WOMB, WOULD YOU IGNORE IT?  1 = Yes  0 = No  99 = Don't know |
| Q 32. IF YOU NOTICED IN YOUR WIFE A SYMPTOM COMING FROM THE CERVIX OR MOUTH OF WOMB, WOULD YOU  DISCUSS IT WITH HER?  1 = Yes  0 = No  99 = Don't know |

| Q 32. IF YOU NOTICED IN YOUR DAUGHTER-IN-LAW A SYMPTOM COMING FROM THE CERVIX OR MOUTH OF YOUR  WOMB, WOULD YOU DISCUSS IT WITH HER?  1 = Yes  0 = No  99 = Don't know |
| --- |
| Q 33. IF YOU HAD A SYMPTOM COMING FROM YOUR CERVIX OR MOUTH OF YOUR WOMB, WOULD YOU TRY SELF-  MEDICATION, FOR EXAMPLE GET SOME OINTMENT TO APPLY FROM THE LOCAL SUPERMARKET?  1 = Yes  0 = No  99 = Don't know |
| Q 34. IF YOU HAD A SYMPTOM COMING FROM YOUR CERVIX OR MOUTH OF YOUR WOMB, WOULD YOU TELL SOMEONE  CLOSE TO YOU?  1 = Yes  0 = No  99 = Don't know |
| Q 34. IF SHE HAD A SYMPTOM COMING FROM THE CERVIX OR MOUTH OF WOMB, WOULD SHE ASK YOUR ADVICE?  1 = Yes  0 = No  99 = Don't know |
| Q 35. IF YOU HAD A SYMPTOM COMING FROM YOUR CERVIX OR MOUTH OF YOUR WOMB, WOULD YOU VISIT A  TRADITIONAL HEALER?  1 = Yes  0 = No  99 = Don't know |

| Q 35. IF SHE HAD A SYMPTOM COMING FROM THE CERVIX OR MOUTH OF WOMB, WOULD YOU RECOMMEND VISITING  A TRADITIONAL HEALER?  1 = Yes  0 = No  99 = Don't know |
| --- |
| Q 36. [ONLY FOR THOSE WHO SAID "YES" IN QUESTION 35] IF YOU HAD A SYMPTOM COMING FROM YOUR CERVIX OR  WOMB, HOW SOON WOULD YOU VISIT A TRADITIONAL HEALER?  1 = <1 week  2 = 1 week < 1 month  3 = 1 month < 3 months  4 = >= 3 months |
| Q 36. [ONLY FOR THOSE WHO SAID "YES" IN QUESTION 35] IF SHE HAD A SYMPTOM COMING FROM THE CERVIX OR  WOMB, HOW SOON WOULD YOU RECOMMEND VISITING A TRADITIONAL HEALER?  1 = <1 week  2 = 1 week < 1 month  3 = 1 month < 3 months  4 = >= 3 months |
| Q 37. IF YOU HAD A SYMPTOM COMING FROM THE CERVIX OR MOUTH OF THE WOMB, WOULD YOU VISIT A  PHARMACY/ CLINIC/ HEALTH CENTRE/ HOSPITAL?  1 = Yes  0 = No  99 = Don't know |
| Q 37. IF SHE HAD A SYMPTOM COMING FROM THE CERVIX OR MOUTH OF THE WOMB, WOULD YOU RECOMMEND  VISITING A PHARMACY/ CLINIC/ HEALTH CENTRE/ HOSPITAL?  1 = Yes  0 = No  99 = Don't know |

| Q 38.[ONLY FOR THOSE WHO SAID 'YES' IN QUESTION 37] IF YOU HAD A SYMPTOM COMING FROM YOUR CERVIX OR  WOMB, HOW SOON WOULD YOU VISIT THE PHARMACY/CLINIC/HEALTH CENTRE/HOSPITAL?  5 = Never  1 = < 1 week  2 = 1 week < 1 month  3 = 1 month < 3 months  4 = >=3 months |
| --- |
| Q 38.[ONLY FOR THOSE WHO SAID 'YES' IN QUESTION 37] IF SHE HAD A SYMPTOM COMING FROM THE CERVIX OR  WOMB, HOW SOON WOULD YOU RECOMMEND VISITING THE PHARMACY/ CLINIC/ HEALTH CENTRE/ HOSPITAL?  5 = Never  1 = < 1 week  2 = 1 week < 1 month  3 = 1 month < 3 months  4 = >=3 months |
| SECTION 5: CONFIDENCE SKILLS AND BEHAVIOR IN RELATION TO A CERVICAL CANCER SIGN/SYMPTOM |
| 39. ARE YOU CONFIDENT THAT YOU WOULD NOTICE A SYMPTOM THAT COULD BE CERVICAL CANCER?  1 = Yes  0 = No  99 = Don't know |
| 39. ARE YOU CONFIDENT THAT YOU COULD GIVE GOOD ADVICE TO YOUR WIFE CONCERNING CERVICAL CANCER?  1 = Yes  0 = No  99 = Don't know |

| 39. ARE YOU CONFIDENT THAT YOU COULD GIVE GOOD ADVICE TO YOUR DAUGHTER-IN-LAW CONCERNING CERVICAL  CANCER?  1 = Yes  0 = No  99 = Don't know |
| --- |
| Q 40. HAVE YOU EVER BEEN TO SEE A NURSE OR CLINICAL OFFICER OR DOCTOR ABOUT A SYMPTOM THAT MADE YOU  THINK SOMETHING WAS WRONG, LIKE A SYMPTOM OF CERVICAL CANCER?  1 = Yes  0 = No  99 = Not noticed any symptoms or signs |
| Q 40. HAVE YOU EVER ACCOMPANIED YOUR WIFE TO SEE A NURSE OR CLINICAL OFFICER OR DOCTOR ABOUT A  SYMPTOM THAT COULD BE CERVICAL CANCER?  1 = Yes  0 = No  99 = Not noticed any symptoms or signs |
| Q 40. HAVE YOU EVER ACCOMPANIED YOUR DAUGHTER-IN-LAW TO SEE A NURSE OR CLINICAL OFFICER OR DOCTOR  ABOUT A SYMPTOM THAT COULD BE CERVICAL CANCER?  1 = Yes  0 = No  99 = Not noticed any symptoms or signs |
| Q 41. HAVE YOU EVER BEEN TO SEE A TRADITIONAL HEALER ABOUT A SYMPTOM THAT MADE YOU THINK SOMETHING  WAS WRONG, LIKE A SYMPTOM OF CERVICAL CANCER? (  1 = Yes  0 = No  99 = Not noticed any symptoms or signs |
| Q 41. HAVE YOU EVER ACCOMPANIED YOUR WIFE TO SEE A TRADITIONAL HEALER ABOUT A SYMPTOM THAT COULD BE  CERVICAL CANCER?  1 = Yes  0 = No  99 = Not noticed any symptoms or signs |

| Q 41. HAVE YOU EVER ACCOMPANIED YOUR DAUGHTER-IN-LAW TO SEE A TRADITIONAL HEALER ABOUT A SYMPTOM  THAT COULD BE CERVICAL CANCER?  1 = Yes  0 = No  99 = Not noticed any symptoms or signs |
| --- |
| SECTION 6: BARRIERS TO SEEKING MEDICAL HELP |
| Q 6A. "WOULD ANY OF THE FOLLOWING REASONS MAKE IT DIFFICULT FOR YOU TO SEE THE NURSE OR CLINICAL OFFICER OR DOCTOR IF YOU NOTICED A SYMPTOM OR SIGN WHICH YOU THINK MAY BE SERIOUS, FOR EXAMPLE A CHANGE IN THE MOUTH OF YOUR WOMB OR CERVIX THAT COULD BE CANCER?" |
| Q 42. I WOULD FIND IT DIFFICULT TO GO FOR MEDICAL HELP BECAUSE I WOULD BE WORRIED ABOUT WASTING THE  NURSE/CLINICAL OFFICER/DOCTOR'S TIME  1 = Agree  0 = Disagree  99 = Don't know |
| Q 42. I WOULD FIND IT DIFFICULT TO ADVICE MY WIFE TO GO FOR MEDICAL HELP BECAUSE I WOULD BE WORRIED  ABOUT WASTING THE NURSE/CLINICAL OFFICER/DOCTOR'S TIME.  1 = Agree  0 = Disagree  99 = Don't know |
| Q 42. I WOULD FIND IT DIFFICULT TO ADVICE MY DAUGHTER-IN-LAW TO GO FOR MEDICAL HELP BECAUSE I WOULD BE  WORRIED ABOUT WASTING THE NURSE/ CLINICAL OFFICER/ DOCTOR'S TIME.  1 = Agree  0 = Disagree  99 = Don't know |

| Q 43. I WOULD FIND IT DIFFICULT TO GO FOR MEDICAL HELP BECAUSE I WOULD BE WORRIED ABOUT WHAT THE  NURSE/CLINICAL OFFICER/DOCTOR MIGHT FIND WRONG.  1 = Agree  0 = Disagree  99 = Don't agree |
| --- |
| Q 43. I WOULD FIND IT DIFFICULT TO ADVICE MY WIFE TO GO FOR MEDICAL HELP BECAUSE I WOULD BE WORRIED  ABOUT WHAT THE NURSE/CLINICAL OFFICER/DOCTOR MIGHT FIND WRONG.  1 = Agree  0 = Disagree  99 = Don't agree |
| Q 43. I WOULD FIND IT DIFFICULT TO ADVICE MY DAUGHTER-IN-LAW TO GO FOR MEDICAL HELP BECAUSE I WOULD BE  WORRIED ABOUT WHAT THE NURSE/ CLINICAL OFFICER/ DOCTOR MIGHT FIND WRONG.  1 = Agree  0 = Disagree  99 = Don't agree |
| Q 44. I WOULD FIND IT DIFFICULT TO GO FOR MEDICAL HELP BECAUSE I WOULD BE WORRIED ABOUT WHAT TESTS THE  NURSE/CLINICAL OFFICER/DOCTOR MIGHT DO.  1 = Agree  0 = Disagree  99 = Don't know |
| Q 44. I WOULD FIND IT DIFFICULT TO ADVICE MY WIFE TO GO FOR MEDICAL HELP BECAUSE I WOULD BE WORRIED  ABOUT WHAT TESTS THE NURSE/CLINICAL OFFICER/DOCTOR MIGHT DO.  1 = Agree  0 = Disagree  99 = Don't know |

| Q 44. I WOULD FIND IT DIFFICULT TO ADVICE MY DAUGHTER-IN-LAW TO GO FOR MEDICAL HELP BECAUSE I WOULD BE  WORRIED ABOUT WHAT TESTS THE NURSE/ CLINICAL OFFICER/ DOCTOR MIGHT DO.  1 = Agree  0 = Disagree  99 = Don't know |
| --- |
| Q 45. I WOULD FIND IT DIFFICULT TO GO FOR MEDICAL HELP BECAUSE I AM TOO BUSY OR HAVE OTHER THINGS TO  WORRY ABOUT.  1 = Agree  0 = Disagree  99 = Don't know |
| Q 45. I WOULD FIND IT DIFFICULT THAT MY WIFE GOES FOR MEDICAL HELP BECAUSE I AM TOO BUSY OR HAVE OTHER  THINGS TO WORRY ABOUT.  1 = Agree  0 = Disagree  99 = Don't know |
| Q 45. I WOULD FIND IT DIFFICULT THAT MY DAUGHTER-IN-LAW GOES FOR MEDICAL HELP BECAUSE I AM TOO BUSY OR  HAVE OTHER THINGS TO WORRY ABOUT.  1 = Agree  0 = Disagree  99 = Don't know |
| Q 46. I WOULD FIND IT DIFFICULT TO GO FOR MEDICAL HELP BECAUSE IT TAKES TOO LONG TO BE SEEN AT THE  CLINIC/HEALTH CENTRE.  1 = Agree  0 = Disagree  99 = Don't know |
| Q 46. I WOULD FIND IT DIFFICULT TO ADVICE MY WIFE TO GO FOR MEDICAL HELP BECAUSE IT TAKES TOO LONG TO BE  SEEN AT THE CLINIC/HEALTH CENTRE.  1 = Agree  0 = Disagree  99 = Don't know |

| Q 46. I WOULD FIND IT DIFFICULT TO ADVICE MY DAUGHTER-IN-LAW TO GO FOR MEDICAL HELP BECAUSE IT TAKES TOO  LONG TO BE SEEN AT THE CLINIC/ HEALTH CENTRE.  1 = Agree  0 = Disagree  99 = Don't know |
| --- |
| Q 47. I WOULD FIND IT DIFFICULT TO GO FOR MEDICAL HELP BECAUSE I HAVE NO MONEY FOR TRANSPORT OR THE  CLINIC/HEALTH CENTRE CHARGES.  1 = Agree  0 = Disagree  99 = Don't know |
| Q 47. I WOULD FIND IT DIFFICULT TO ADVICE MY WIFE TO GO FOR MEDICAL HELP BECAUSE I HAVE NO MONEY FOR  TRANSPORT OR THE CLINIC/HEALTH CENTRE CHARGES.  1 = Agree  0 = Disagree  99 = Don't know |
| Q 47. I WOULD FIND IT DIFFICULT TO ADVICE MY DAUGHTER-IN-LAW TO GO FOR MEDICAL HELP BECAUSE I HAVE NO  MONEY FOR TRANSPORT OR THE CLINIC/ HEALTH CENTRE CHARGES.  1 = Agree  0 = Disagree  99 = Don't know |
| Q 48. I WOULD FIND IT DIFFICULT TO GO FOR MEDICAL HELP BECAUSE I WOULD NOT FEEL CONFIDENT ABOUT  TALKING ABOUT MY SYMPTOMS.  1 = Agree  0 = Disagree  99 = Don't know |

| Q 48. I WOULD FIND IT DIFFICULT THAT MY WIFE WOULD GO FOR MEDICAL HELP BECAUSE I WOULD NOT FEEL  CONFIDENT THAT SHE TALKS ABOUT SUCH DISEASE THAT COULD AFFECT ME AS WELL.  1 = Agree  0 = Disagree  99 = Don't know |
| --- |
| Q 48. I WOULD FIND IT DIFFICULT THAT MY DAUGHTER-IN-LAW WOULD GO FOR MEDICAL HELP BECAUSE I WOULD NOT  FEEL CONFIDENT THAT SHE TALKS ABOUT SUCH DISEASE THAT COULD AFFECT ME AS WELL  1 = Agree  0 = Disagree  99 = Don't know |
| Q 49. I WOULD FIND IT DIFFICULT TO GO FOR MEDICAL HELP BECAUSE I HAVE HAD A BAD EXPERIENCE IN THE  CLINIC/HEALTH CENTRE IN THE PAST.  1 = Agree  0 = Disagree  99 = Don't know |
| 49. I WOULD FIND IT DIFFICULT TO ADVICE MY WIFE TO GO FOR MEDICAL HELP BECAUSE I HAVE HAD A BAD  EXPERIENCE IN THE CLINIC/ HEALTH CENTRE IN THE PAST.  1 = Agree  0 = Disagree  99 = Don't know |
| Q 49. I WOULD FIND IT DIFFICULT TO ADVICE MY DAUGHTER-IN-LAW TO GO FOR MEDICAL HELP BECAUSE I HAVE HAD A  BAD EXPERIENCE IN THE CLINIC/ HEALTH CENTRE IN THE PAST.  1 = Agree  0 = Disagree  99 = Don't know |

| Q 50. I WOULD FIND IT DIFFICULT TO GO FOR MEDICAL HELP BECAUSE I WOULD FEEL EMBARRASSED.  1 = Agree  0 = Disagree  99 = Don't know |
| --- |
| Q 50. I WOULD FIND IT DIFFICULT TO ADVICE MY WIFE TO GO FOR MEDICAL HELP BECAUSE I WOULD FEEL  EMBARRASSED IF SHE TALKS ABOUT SUCH DISEASES.  1 = Agree  0 = Disagree  99 = Don't know |
| Q 50. I WOULD FIND IT DIFFICULT TO ADVICE MY DAUGHTER-IN-LAW TO GO FOR MEDICAL HELP BECAUSE I WOULD  FEEL EMBARRASSED IF SHE TALKS ABOUT SUCH DISEASES.  1 = Agree  0 = Disagree  99 = Don't know |
| Q 51. I WOULD FIND IT DIFFICULT TO GO FOR MEDICAL HELP BECAUSE THE NURSE/CLINICAL OFFICER/DOCTOR WOULD  NOT UNDERSTAND MY LANGUAGE OR CULTURE.  1 = Agree  0 = Disagree  99 = Don't know |
| Q 51. I WOULD FIND IT DIFFICULT TO ADVICE MY WIFE TO GO FOR MEDICAL HELP BECAUSE THE NURSE/ CLINICAL  OFFICER/ DOCTOR WOULD NOT UNDERSTAND MY LANGUAGE OR CULTURE.  1 = Agree  0 = Disagree  99 = Don't know |
| Q 51. I WOULD FIND IT DIFFICULT TO ADVICE MY DAUGHTER-IN-LAW TO GO FOR MEDICAL HELP BECAUSE THE NURSE/  CLINICAL OFFICER/ DOCTOR WOULD NOT UNDERSTAND MY LANGUAGE OR CULTURE.  1 = Agree  0 = Disagree  99 = Don't know |

| Q 52. I WOULD FIND IT DIFFICULT TO GO FOR MEDICAL HELP BECAUSE MY HUSBAND/PARTNER OR FAMILY MEMBER  WOULD NOT ALLOW ME TO GO.  1 = Agree  0 = Disagree  99 = Don't know |
| --- |
| Q 52. I WOULD FIND IT DIFFICULT TO ADVICE MY WIFE TO GO FOR MEDICAL HELP BECAUSE OTHER FAMILY MEMBERS  WOULD NOT AGREE.  1 = Agree  0 = Disagree  99 = Don't know |
| Q 52. I WOULD FIND IT DIFFICULT TO ADVICE MY DAUGHTER-IN-LAW TO GO FOR MEDICAL HELP BECAUSE OTHER  FAMILY MEMBERS WOULD NOT AGREE.  1 = Agree  0 = Disagree  99 = Don't know |
| Q 53. I WOULD FIND IT DIFFICULT TO GO FOR MEDICAL HELP BECAUSE I THINK THAT 'IF I HAVE A DISEASE LIKE CANCER  THERE IS NO USE FOR THE NURSE/ CLINICAL OFFICER/DOCTOR AND I WILL DIE ANYWAY'.  1 = Agree  0 = Disagree  99 = Don't know |

| Q 53. I WOULD FIND IT DIFFICULT TO ADVICE MY WIFE TO GO FOR MEDICAL HELP BECAUSE I THINK THAT 'IF SHE HAS A  DISEASE LIKE CANCER THERE IS NO USE FOR THE NURSE/ CLINICAL OFFICER/ DOCTOR AND SHE WILL DIE ANYWAY'.  1 = Agree  0 = Disagree  99 = Don't know |
| --- |
| Q 53. I WOULD FIND IT DIFFICULT TO ADVICE MY DAUGHTER-IN-LAW TO GO FOR MEDICAL HELP BECAUSE I THINK THAT  'IF SHE HAS A DISEASE LIKE CANCER THERE IS NO USE FOR THE NURSE/ CLINICAL OFFICER/ DOCTOR AND SHE WILL DIE  ANYWAY'.  1 = Agree  0 = Disagree  99 = Don't know |
